# Shoah pornography: the Stalag phenomenon in Israel during the 1960s

**DOI:** 10.3389/fpsyg.2025.1380813

**Published:** 2025-06-25

**Authors:** Jasmin Spiegel, Arne Dekker, Peer Briken, Anika Gomille, Martin Rettenberger

**Affiliations:** ^1^Hebrew University of Jerusalem, Jerusalem, Israel; ^2^University Medical Center Hamburg-Eppendorf, Hamburg, Germany; ^3^University of Siegen, Siegen, Germany; ^4^Centre for Criminology, Wiesbaden, Germany

**Keywords:** Stalag, Shoah, Pornography, Trauma, Expert interviews

## Abstract

The ‘Stalags’ (Orig. ‘Stalagim,’ Abbr. German = ‘Stammlager’) are soft pornographic booklets portraying a sadomasochistic plot at the time of Shoah. The aim of this study is to investigate the psychological and sociocultural causes of this phenomenon in Israel during the 1960s. To this end, five expert interviews were conducted and evaluated using a qualitative content analysis. In total, six categories were extracted, which provided the basis for a discussion of the popularity and spread of Stalags during the Adolf Eichmann trial in Israel. First, reversal of hatred of antisemitic images of Jews; second, sexuality as a political category; third, defense of traumatic guilt by sexualization; fourth, sexuality as a practical category using feelings of power leading to sexual arousal; fifth, reflection of the new idea of masculinity (‘The Sabra’) in society; and sixth, the influence of American Hollywood cinema on aesthetics and plot. These categories were enriched using psychoanalytic theory.

## Introduction

*To paraphrase Hannah Arendt’s dictum, in the Stalags evil is no longer banal—it is exciting. As such, these narratives constitute a counter-narrative to the story of destruction—heroism, victory and sex as the ultimate triumph of libido over death* (Pinchevsky and Brand, 2007, 395).

In 2008, Israeli film director Libsker produced the documentary film ‘Holocaust and Pornography.’ He portrayed the until then rather unknown occurrence of the Stalags, which are pornographic booklets written by Israeli authors, including the children of survivors of the Shoah, in the 1960s in Israel. The writers sought to give the impression that the booklets were Hebrew translations of US-American or British reports of Stalag prisoners by writing in a roundabout way and using English pseudonyms like Mike Baden or Ralph Butcher (Pinchevsky and Brand, 2007). Although etiologies of deviant sexual interests such as sadomasochism have been developed extensively in the literature (Schippers, 2024), there seems to be something specific about the historical-social context of this sadomasochistic genre that explains the phenomena beyond the field of traditional clinical psychopathology: The plot of the Stalag pulp fiction is generally constructed as follows: a male hero, usually a British soldier, is taken to Germany during the Second World War and—after getting into war captivity—is put into a ‘Stalag’ camp, resembling a National Socialist labor camp. He and other prisoners are tortured and sexually harassed there by attractive ‘Aryan’ female attendants with blonde hair, esthetic shapes, and curvy breasts in tight Schutzstaffel (SS) uniforms. These actions are accompanied by masochistic arousal on behalf of the prisoners. Then, a turning point occurs at which the prisoner can flee the camp and take revenge by raping and killing female attendants who had previously tortured him. The first booklets were published at the beginning of the court process against Adolf Eichmann (SS senior assault unit leader) in 1961, and over 80,000 copies were sold, with decreasing sales volumes in the following years (Libsker, 2008). The Stalags were an easily acquired mass pornography in sexually repressive Israel of the 1960s. Initially, they were sold at kiosks and bookstores and privately distributed in military canteens or schools. Despite widespread circulation within Israel, no archival evidence has confirmed its systematic distribution abroad. The identity of the authors of the Stalags was unknown at that time, as they used pseudonyms to pretend that they were writers from outside Israel, such as Israeli author Eli Keidar, who used the pseudonym Mike Baden. At the end of the Eichmann trial in 1962, public interest decreased, and the last editions sold fewer than 100 copies, indicating a temporarily limited phenomenon. Additionally, some pornography was banned by the government because of the portrayal of extreme violence (e.g., ‘I was Captain Schulz’s Private Bitch,’ the first pornography trial in Israel, 1962–1963). Together with the publisher Yitshak Gutman and others involved in the production in publishing houses such as Eshet Press, the author Eli Keidar was prosecuted after his trial (Leket-Mor, 2011, p.30). The Stalag phenomenon quickly faded, followed by a general subsiding of the pulp industry in the 1970s in favor of television broadcasting and a change in the political party at that time that led to a “normalization” and “stratification” of the literary system (ibid., p.36). Despite the cultural and psychological implications of the Stalag phenomenon, research on trauma studies and Holocaust historiography has largely neglected its analysis, with existing work often being limited to clinical or cultural readings. The marginalization and taboo surrounding the Stalags` content suggest an inability to integrate the ambivalent fantasies of victimhood, power, and sexuality they embodied. This study addresses the need for an interdisciplinary framework that bridges psychoanalysis, media representation, and memory politics to understand how Stalags function as sites for post-Holocaust trauma negotiations. The main research question aims to provide answers to their limited regional popularity in the 1960s.

### The genre

Previous researchers assigned the Stalags to the media sector of so-called Sadiconazista or Deathcamp pornography, which started in the 1960s in Italy and included various occurrences ranging from soft pornographic portrayals in image and content to the rape of physically and mentally disabled persons in concentration camps (Stiglegger, 2011). Sexual sadistic behavior and masochistic desire seem to be the major common denominators of these movies and books, which played with historical facts, including places, persons, or events in a storyline set in the last days of the liberation of Germany by victorious powers. Unmistakably, the authors and producers attempted to establish a romantic ‘downfall’ scene. The protagonists of the stories are despots, cold-blooded party functionaries, seductive SS officers, and kapos (prisoner functionaries) on the perpetrators’ side, facing rebellious victims who take revenge on the enemy at the end of the storyline. The phenomenon of the Stalags occurred when the survivors of the Shoah had stabilized themselves physically and existentially, although no psychological working-through of traumatic experiences had occurred. The Stalags could be seen as ‘psychic retreats,’ as first described by Steiner (1993), or ‘collated internal objects’ (Khan, 1965)—a perversely organized space wherein it is possible to retreat from a frightening reality; during this process, the (perverse) fantasies become part of an inner reality. It is important to note that the term “perverse” is used in a psychoanalytical fashion to describe any form of deviating sexual behavior, not suggesting any moral judgment of this behavior or a pre-given natural order, as the everyday language of the term implies. For Freud, human sexuality has been polymorphous perverse since the beginning. We are interested in the psychic and social functions of the perverse elements covered in Stalag pulp fiction.

### Historical context

By the time of the arrest of Adolf Eichmann, 16 years after the end of the Second World War, the first contact with the Shoah in Israeli society had occurred. This contact has been described as the realization of a psychotic cosmos in concentration camps (Klein, 2012a). The Eichmann trial confronted Israeli society with the until-then-long-suppressed dread of the Shoah. It occurred in a nationally and politically complicated situation (Krause, 2002). After declaring independence in 1948 and defending against pan-Arabic attacks, the last military confrontation occurred with Egypt during the 1956 Suez War. The sociopolitical situation of Israel confronted its citizens with the challenge of forming individual and national identities. Diverging concepts of daily culture and statehood, the handling of Shoah survivors, the integration of Jews from the Middle and Near East, and persistent controversies between religious and secular Jews were negotiated domestically, while within foreign policy, past threats to Jewish life did not lose their power.

### Previous explanations

Previously published studies on the Stalags explain their high resonance, particularly among young recipients during and after the Eichmann trial—beyond the thrill of the sexually illicit—as an intergenerational negotiation of past traumatic experiences. Pulp Fiction can thus be understood as a ‘Post-traumatic Pornography Syndrome’ (Bleimling and Kind, 2015): documented transformations of the suffered and survived traumatizations through the Shoah.

By analyzing works such as Ka-Tzetnik’s *House of Dolls*, Sidney Lumet’s *The Pawnbroker*, Liliana Cavani’s *The Night Porter*, and William Styron’s *Sophie’s Choice*, Hoffman and Gross (2021) demonstrate how the suffering body is both a symbol of victimization and desire and how these obscene narratives can blur the lines between memorialization and exploitation. Furthermore, Stalags constitute a text upon which Israeli youth negotiated issues of power and identity vis-à-vis both their parents’ generations and the Zionist ideology. The sadomasochistic configuration served as a platform for reworking conflicting tendencies within Israeli identity politics, intensified by the Eichmann trial (Pinchevsky and Brand, 2007). Stalags can be seen as a negotiation of identity issues in the 1960s, a time when Israel was metaphorically stuck in adolescence and needed to form a new identity through an altercation between inner and outer objects. Hence, parents are perceived as weak and/or victims, in blatant contrast to the new peer group, the so-called Sabras, i.e., strong, potent, and Zionist men (ibid.). As in adolescence, the teenager separates from his childhood (the traumatic past of the Shoah) by means of identification (with the new peer group in society) to obtain separation and individuation from inner primary objects. The importance of the attachment-individuation process has been prominent in the work of psychoanalytic authors such as Diamond (2005), who sketches a model of masculine gender identity as an answer to the interplay between masculine and feminine identifications (thus distancing himself from Freud’s initial model of disidentification with the mother). Furthermore, Fonagy (1999) asserted that violence against women can be linked to attachment relationships, specifically, disorganized attachment patterns in infancy. As Stoller (1975, xi) points out, ‘perversion is the result of an essential interplay between hostility and sexual desire; one connotation of this term hints at the need of a society to provide “scapegoats” who liberate the rest of us in that they serve as the objects of our own unacceptable and projected perverse tendencies.’ Through his clinical work, he found perversion to be ‘a way of coping with threats to one’s gender identity, that is, one’s sense of masculinity and femininity’ (1975, xii). These theoretical assumptions could support our understanding of the Stalag phenomenon against the background of gender issues previously outlined in the 1960s in Israel. However, in our opinion, previous explanations, which are grounded in conceptual analysis, are not sufficient because individual psychological interpretations are given priority, and collective processes, as well as sociocultural influences, have not been sufficiently examined. Starting with Freud, who already explained cultural products, such as mass phenomena, with individual psychological processes, modern theorists agree that psychoanalysis is mainly a pathoanalysis of human existence with the consequence “that we can study certain cultural products in which important aspects of human existence are expressed from the perspective of these pathologies” (Westerink, 2020, 584). Contemporary Holocaust studies, particularly those by scholars such as Rothberg (2009) and Hirsch (2012), provide their concepts of multidirectional memory and postmemory critical frameworks for situating the Stalag phenomenon not merely as individual psychological artifacts but as contested sites within transgenerational and transnational memory cultures shaped by visual media, taboo, and political silences. The aim of the present study was to obtain a comprehensive explanatory model of the Stalags by examining different expert opinions from the field of individual clinical psychology as well as from cultural science and history and enriching their argumentation with the above-mentioned existing theoretical knowledge.

## Materials and methods

Five expert interviews were conducted in 2014 by the second and last authors using semi-structured interview guidelines in German with interviewees, which were qualitatively evaluated by the first author. As the term ‘expert interview’ assumes, the prerequisite for an expert is his or her specialized knowledge, which is distinct from that of a layperson. To represent an interdisciplinary perspective on the phenomenon, the interviewees were chosen because of their specialized scientific experience, which ranges from historical and sociocultural perspectives to psychoanalysis, forensic, and sexological perspectives. Specific criteria for selection of the experts were previous knowledge about etiology and contemporary models of deviant sexual behavior from an individual psychological as well as a sociocultural perspective, advanced academic qualifications in relevant fields such as psychology, criminology, forensic psychology, and history; practical experience working with a wide range of individuals exhibiting deviant sexual behaviors; and willingness to discuss a potentially controversial issue from a scientific and ethical perspective. To prepare for the interviews, a video recording of Libsker’s (2008) documentary film was sent to participants beforehand. The German interviewees had limited prior knowledge of the phenomenon, whereas the Israeli interviewee was well informed because of his own biography as a descendant of German Jews growing up in Israel and as a witness to the Stalags as a teenager in the 1960s. The duration of the semi-structured interviews varied between one and two hours, beginning with an open introductory question about the expert’s individual contact with the Stalags and proceeding with guideline-supported questions about the reasons for their popularity. We are also aware that an independent expert’s perspective is not free from entanglements and projections of the material, which will be considered in the presentation of the categories. Transcriptions of the audio recordings functioned as the database for analysis, based on Mayring’s (2000) qualitative content analysis approach. This methodological approach was chosen to analyze the material because the concrete way of asking questions already provides a certain pattern of thoughts and answers. It gives the opportunity to promote both inductive category development and deductive category application. The deductive excerption was fruitful because of the former theoretical engagement with the topic, which in turn facilitated the inductive excerption. All interviews were analyzed in a successive manner following the methodological guidelines of qualitative content analysis (Mayring, 2000) to divide the material into its essential ingredients (formulated as content analytical units). After the theoretical justification and specification of interest in the Stalag phenomenon, materials were selected and characterized. A reference interview was chosen because of the richness of its explanations. The other four interviews were used to confirm the reference categories and mark additional categories, thus confirming, contrasting, and expanding the emerging categories. In total, four devising loops were carried out by an independent evaluator who was not involved in the interview, solely in the excerption of the categories. This iterative coding process ensured interpretive depth and thematic saturation. The small sample size was found to be appropriate, as our participants were highly knowledgeable in their respective fields, and the study aim was limited to the Stalag phenomenon.

## Results

**Figure 1:**
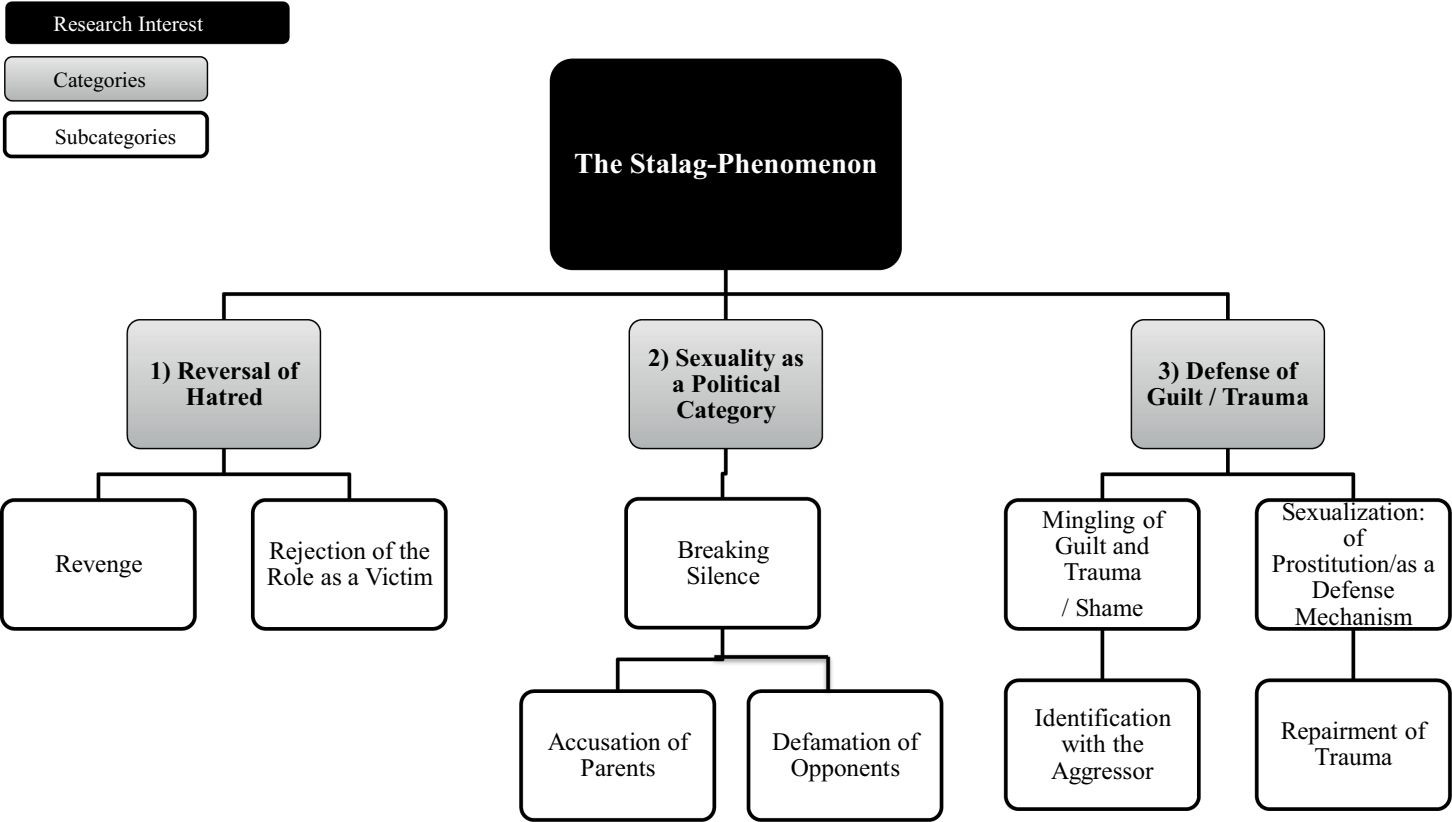
Categories and sub-categories which are deemed as suitable to describe the Stalag-phenomen in the content area ‘Family’.

**Figure 2:**
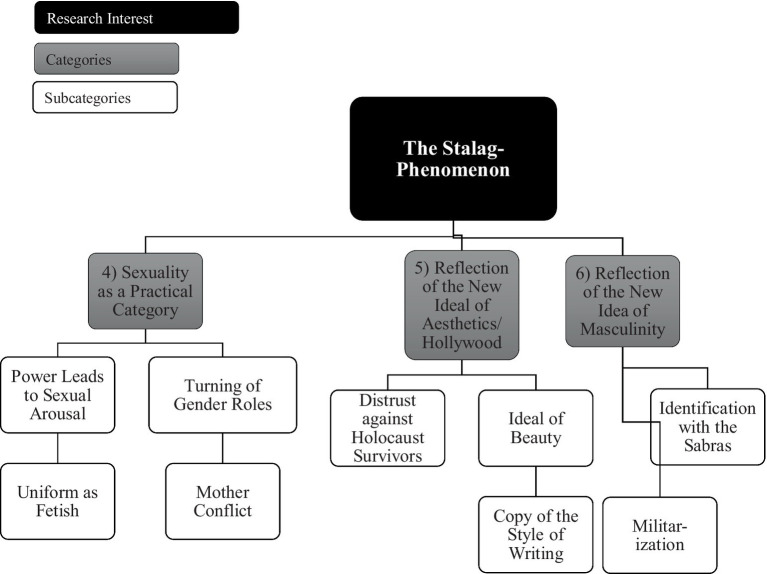
Categories and sub-categories which are deemed as suitable to describe the Stalag-phenomen in the content area ‘Society’.

The main results are presented in the following two figures (see Figure 1, ‘Family’ and Figure 2, ‘Society’), including the six extracted main categories as well as their allocated subcategories, illustrated by text passages of the transcript of the main reference interview with the historian interviewee. In addition to presenting subjective insights derived from the qualitative content analysis, specific shifts and dynamics in the speaker’s position during the reference interview were analyzed. In this regard, the expert himself was experienced in answering questions on the borderline between being an eyewitness and an expert historian. Because the other four German interviewees did not have any personal experience with the topic, the biographical access of the Israeli interviewee can be considered an important additional value and is the main reason why this interview was chosen as the reference interview. His interview began with a brief statement of his personal involvement, describing his contact with pulp fiction as a pupil at a Jewish Orthodox school as ‘very intriguing.’ Afterwards, he addresses his role as a historian and ‘his duty’ to find out why the Stalags had such limited temporal relevance. Throughout the interviews, this change in perspective was maintained, especially regarding the topic of sexuality, where he took measures to maintain a professional distance from the phenomenon. Accordingly, the two extracted categories, ‘Family’ and ‘Society,’ mirror these two perspectives. On the one hand, the category ‘Family’ includes intrapersonal feelings such as revenge, shame, and repair connected to the experience of the survival of one’s own parents and their traumatic experiences in the Shoah, which may reflect the interviewer’s personal experiences with this topic (born as a son of German Jews emigrating to Palestine in the 1930s). On the other hand, the category ‘Society,’ which contains societal influences like ideals of beauty and masculinity as well as obeying the social convention of identifying with the militarization of the State of Israel, could resonate with the interviewer’s expert perspective when talking about his research field of Israeli and German history.

### Category 1: reversal of hatred

This category refers to hatred arising from hostile and antisemitic images of Jews during the Shoah period. These images were reversed in the Stalag booklets to both take revenge on the perpetrators and reject the introjected parental role of being fragile victims. Furthermore, from a societal perspective, along with the Eichmann trial, a historical turn took place: previously chased Jews were now chasers themselves. It can be assumed that this new dominant revenge-taking position was mirrored in the Stalags.

This interpretation is based on the functional-dynamic conceptualization of perversion and can probably be regarded as one of the most authoritative concepts in the psychoanalytic community. For example, Stoller (1975) describes the hostile part of perversion ‘as the erotic form of hatred, [which] serves to convert childhood trauma to adult triumph’ (4) and further states that ‘perversion is the reliving of actual historical sexual trauma aimed precisely at one’s sex or gender identity’ (6). Stoller’s prerequisites for the classification of perversion (i.e., as primarily driven by hostility, revenge, triumph, and a dehumanized object) can be recognized in the Stalag pulp fiction: Like every other perversion, this kind of pornography is also based on a fantasized act of revenge. It stands to reason that the experienced inferiority of the children of survivors in the 1960s in Israel was transformed into feelings of triumph, dominance, and superiority in the Stalags by the regularly painted picture of revenge on the warden. The perverse act lies in the desire to harm the object of excitement (SS-uniform women) together with a feeling of triumph. Of course, Stoller’s view is taken here into an extreme version by reliving the deformation and dehumanization of the sexualized object, as has been experienced in the past by Shoah traumatizations. This process of taking revenge must be understood as a collective one because being a survivor is internalized as a group identity.

Subcategories:

1a) Aggressive reversal of Jewish hatred and sexual images of Jews in antisemitism.

1b) Revenge.

1c) Rejection of the (introjected/parental) victim role.

**Table 1:** 

Text	Subcategory
You can see all those projections of Jews as libidinous or female, sensual, false, etc. They also experienced another inversion. I have seen this as an attempt to keep those projections away to convert them into aggressive conversions.	1a) Reversal of Jewish hatred and sexual images of Jews in antisemitism
The Nazis strongly transferred ‘Germania’ to the German female body raped by Jews. Furthermore, the Jew says, ‘You were alleging that we raped your women. NOW we really do it!’ in the manner of speaking to convert it into an activity, which has always been a presumption.	1b) Revenge
What has been connected to the Germans and to your own victim role, from which you want to escape, that you want to get rid of?	1c) Rejection of the (introjected/ parental) victim role

### Category 2: sexuality as a political category—conflict between generations

The consumption of the Stalags was understood as a mainly inter-familial revolutionary act by the young second generation born immediately after the Shoah. Due to the historical entanglement of pornography with the Shoah, the genre disrupted the prevailing taboos and silences surrounding traumatic experiences, intensifying public discourse. Accordingly, pornography was used as both a tool and a door opener for a political debate. Simultaneously, as the opponents, the Nazis were defamed as feminine, weak, and ambivalent by portraying themselves as female attendants in the Stalags, and the adult generation (the first generation of Shoah survivors) was provoked until they were accused and degraded. Tapper (2013) describes the Stalags with their carefully edited scenarios (such as not showing aggression against Jews in concentration camps or by staging the male protagonists as British soldiers) as a part of a mythologization (in the sense of blurring historical facts) of the Nazi past. Although the Stalags are staged exclusively on an individual sadomasochistic level, which might lead to a depolarization of the trauma of the Shoah, experts argue that consumers were starting to deal with political questions such as guilt and responsibility in an inner-family context.

Subcategories:

2a) Breaking silence/taboo.

2b) Accusation/degradation/provocation of parents.

2c) Defamation of the opponent as feminine/weak/ambivalent.

**Table 2:** 

Text	Subcategory
It had to do more with the Holocaust and with what has been tabooed against which you wanted to rebel.	2a) Breaking silence/taboo
I do not even know if young people want to provoke in a way that their parents, as victims, will start to talk finally. I think they were more interested in the discourse: you behaved in a disgraceful way, you went into the Konzentrationslager (KZs) almost voluntarily. It was more an accusatory discourse.	2b) Accusation/degradation/ provocation of parents
The defamation of the opponent via sexual images—those function a lot with feminization. The opponent is effeminate and false, like all women, and so on; he is also ambivalent and indistinct.	2c) Defamation of the perpetrator as feminine/weak/ambivalent

### Category 3: defense of guilt/trauma

A common phenomenon known through clinical work with traumatized patients is the survivor’s suffering from feeling guilty. ‘In the face of their cumulative losses and these inescapable yet impossible choices, of the mental mechanisms available to adapt or defend against their powerlessness, self-blame, and consequential guilt were almost inevitable,’ states a child of survivors (Garwood, 1996, 246). Subcategories 3a) Mingling of guilt and trauma and 3b) Identification with the aggressor/perpetrator can be understood as constituent parts of the same underlying mechanism. As the feeling of guilt is transformed into internal violence, its inner-psychological consequence is a reduction in feelings of guilt. Temporary relief from helplessness through identification with the aggressor creates a feeling of triumph and at the same time breaks the ‘culture of silence’ that accompanied the social trauma of the Shoah. This culture of silence, in the sense of not being able to speak, can be clearly distinguished from the silence of the perpetrators (not wanting to speak), as well as the different associated defense mechanisms. For this reason, it is also understandable why the Stalags hardly found a market in other countries, for example, in Germany, where the real guilt of the perpetrators was negotiated in other forms as per the above-described mechanisms, which provides one answer to why the Stalags were exclusively successfully sold in Israel. Furthermore, subcategory 3c) Shame/Education via the Eichmann trial explains the time limitation of Stalags’ popularity. After the testimonies were brought up in public during the Eichmann trial, the actual horrors of the Shoah could not be ignored in the until-then silent private households of survivors’ families, and the Stalags were read much less.

Subcategories:

3a) Mingling of guilt and trauma.

3b) Identification with aggressor/perpetrator.

3d) Shame/education via the Eichmann Trial—reduced consumption of Stalags.

**Table 3:** 

Text	Subcategory
Well, I mean guilt and trauma are close to each other, right? How quickly a trauma can convert into feelings of guilt. Everyone who survived a trauma asks continuously, “What did I contribute?” Am I guilty myself of what happened to me?	3a) Mingling of guilt and trauma
You can see this with abused children, who start beating others themselves. In this moment, they re-function their own violence, so to speak, to get rid of their guilt feelings, at least for this period of time.	3b) Identification with the aggressor/ perpetrator?
It is known that the Eichmann trial suddenly made clear what really happened and how little the power of decision-making was. From this moment on it turned into something, that inherits a certain barrier of shame.	3c) Shame/education via the Eichmann trial

### Category 4: sexuality as a practical category—power leads to sexual arousal

The underlying mechanism of this category states that, in general, sexually violent materials can lead to feelings of liberation and potency. The consumer of the pornography felt almost as if a real (violent) action was taking place, i.e., liberation from the labor camp and revenge being taken by the act of rape, through which feelings of potency and sexual arousal appeared.

**Table 4:** 

Text	Category
I’d say that violent pornography does not need to be connected with sexuality necessarily. But with feelings of power that arise out of those sexual power phantasies. It was like a liberation of action.	Sexuality as a practical category ➔ power led to sexual arousal

### Category 5: reflection of the new idea of masculinity in society

In Israeli society of the 1960s, the idea of physically strong young Zionist men, ‘Sabras’ (Almog, 2000), as well as the growing militarization of the country, influenced the dominance and importance of a new ideal of forceful and robust masculinity in society, which is represented by the character of the actively revenge-taking British soldier in the Stalags.

Subcategories:

5a) Identification with the new idea of masculinity.

5b) Militarization.

**Table 5:** 

Text	Subcategory
There was a conflict between those Zionists, who came before 33 to Palestine. The young generation identified themselves more with those Zionists, who had everything that the old Jews did not have, namely a body, muscles, and good training that occupied the ground, and so on.	5a) Identification with the new idea of masculinity
That is the discourse of a militarization from the unconscious to a conscious level. At this very moment starts the real discourse: we need to defend ourselves, and we need to build up an army that never permits what happened again.	5b) Militarization

### Category 6: influence of the Hollywood cinema on aesthetics and plot

The ideal of how women and men should look like (strong, muscular, blond, beautiful) and should act according to their gender roles was not only influenced by the inner Israeli ‘Sabras’ idea but also by the world power USA, which was personified by Hollywood cinema. Both a definite ideal of beauty and a certain way of writing and directing were copied in the Stalags to adhere to the standards of Hollywood cinema to increase their popularity.

Subcategories:

6a) Ideal of beauty with respect to women.

6b) Copying the style of writing.

**Table 6:** 

Text	Subcategory
It is probably connected with Hollywood cinema that it had already shaped the concept of the beautiful woman, the images of women that you wanted to join.	6a) Ideal of beauty with respect to women
I really assume that it has been an attempt to lean on cinema anyhow. And cinema at this time was the United States.	6b) Copying the style of writing

In the following, additional subcategories will be briefly presented to provide supplementary information to the categories ‘Family’ and ‘Society.’ Subcategories were extracted from the other four interviews to enrich the main categories of reference interviews.

### Subcategories

#### Subcategory for category 1: reversal of hatred

##### 1d) Uniform as fetish

A detailed description of the Nazi uniforms and boots in the Stalags, which symbolizes their wearers’ power, can be seen as equivalent to a fetish that is sexually arousing. Reiche (2005) describes a fetish as a ‘sexualized transitional object’ (i.e., a transitional object according to Winnicott, 1953). Moreover, the fetish is an object created by the subject, which closes the ‘gap in the self’ (Reiche, 2005, 140). This assumption is in line with Morgenthaler’s concept of the ‘perverse seal’ (1974) to counteract mental disorganization or Greenacre’s (1971) ‘necessary prop,’ which interpreted perversions as a function that ‘closes the gap that creates a missing narcissistic development’ (Morgenthaler, 1974, 1,081). In the case of the Stalags, a possibly traumatizing early childhood relationship constellation (i.e., the silence of the extremely traumatized parents’ generation) could result in a narcissistic gap in the self (i.e., by transforming these traumatic experiences to the subsequent generation), which is ‘bound’ in the perversion and has the function of stabilizing the ego.

#### Subcategories for category 3: defense of guilt/trauma

##### 3d) Sexualization of real/virtual prostitution

Beyond the survivor’s self-accusations and feelings of guilt, they were confronted with distrust and accusations (e.g., the reproach of prostitution of Jewish women in the Third Reich) by Israeli society, so that sexualization reaches beyond a metaphorical level to a real, concrete level of experience. Thus, the underlying psychological process can be understood in terms of imaginative processing of a real threat in pornography.

##### 3e) Sexualization as a defense mechanism/Rescue from death/compensation for trauma

One way of dealing with unbearable emotions is to defer them to bearable emotions. ‘Sexualization is depictions of the Holocaust, (…), stands in direct relation to the portrayal and perception of the perpetrator’s complexity and culpability, therefore functioning to either dismiss the perpetrator’s humanity or to relativize their liability’ (Aretz, 2019, 2). Here, Sexualization is understood as a form of ‘compensating’ or ‘repairing’ the trauma: a rescue and coping attempt against real experienced death and losses during traumatic incidents.

#### Subcategory for category 5: new ideal of masculinity

##### 5c) Distrust of Holocaust survivors in the ‘new’ society

The strong will of the survivors of the Shoah to build up a new home in Israel and ‘forget the traumatic past’ was confronted with reserve and distrust by non-survivors, who lived in Palestine before the Nazi regime and had no traumatic European past. This led to an increased pressure, especially on the children of the survivors, to adopt the existing ideals of serving and defending the country without fear or reluctance as a strong `Sabra.`.

The subsequent categories 7–9 provide more arguments for societal influences beyond the reference category ‘Society’ and were extracted from all five interviews.

#### Category 7: double breaking of taboos (pornography and the perspective of perpetrator)

The popularity of the Stalags stemmed from the excitement connected to breaking two taboos at once: the first emerged from the use of pornography in times of sexually strict morals in society, and the second from taking the perspective of the perpetrator.

**Table 7:** 

Text	Category
On one side there is Nazism; on the other side there is the perpetrator’s perspective. In my opinion, this combination of two taboos is commercially ideal. Well, these are all very widely tabooed elements of sexuality in a sexually very restricted society that can lead to sexual appeal amongst the youth.	Double breaking of taboos (pornography and the perspective of the perpetrator)

### Category 8: technical reasons—no alternative mass pornography/‘sex sells’

Because alternative mass pornography was rarely available at that time, one can argue economically that the supply determined the demand and—more generally speaking—that the often-cited assumption of ‘sex sells’ might be applicable to the explanation of the success of the Stalags.

**Table 8:** 

Text	Category
Well, at these times there was probably no pornographic literature at all.	No alternative mass pornography
Something that deals with sex. The popularized history sells.	Sex sells

### Category 9: deferral of British protectors

The protagonists of the Stalags were seen as mirrors of the political situation of being dependent on a mighty yet ambivalent power: British soldiers as protectors of the new state.

**Table 9:** 

Text	Category
I thought of Israel as an English protectorate. And that it has to do with the fact that the protagonist is fantasized as an Englishman. Protection and oppression.	Deferral of British protectors

## Discussion

This study sought to provide a detailed explanatory model of Stalags by exploring five expert viewpoints from individual clinical psychology, cultural studies, and history, while critically engaging with existing knowledge of the phenomenon. The main insights will be summed up along with the main extracted categories: Trauma, Perversion, Intergenerational Conflicts, and Society. Overall, the marginalization and taboo surrounding the Stalags` content suggest an inability to integrate the ambivalent fantasies of victimhood, power, and sexuality that they embodied.

### Trauma

‘Not only the timing (…), but the very language reveals how the Stalag as a genre constituted an early response to the trauma of the Holocaust’ (Vitiello, 2018, 142). The main extracted categories were *1) reversal of hatred* and *3) defense of guilt/trauma* containing important post-traumatic symptoms, such as dealing with the inner roles of victim and perpetrator, the mingling of guilt and trauma, and the associated shame of the survivor’s guilt. Furthermore, the experts also addressed psychoanalytic defense mechanisms, which can be interpreted as means to ‘repair’ and overcome the trauma. At the same time, these defense mechanisms were accompanied by the described symptoms, such as identification with the aggressor, sexualization, fantasized revenge, and denying their own victim role. Generally, the origin of fantasies expressed in pornography can be seen as an ‘act of revenge’ (Stoller, 1975), so the attraction of the Stalags could be based on the eroticization of hatred (Bleimling and Kind, 2015, 81). This is related to an important post-traumatic function: the defense of one’s own fear of persecution and the reduction of fear and threat against an (inner) persecuting object. The defense mechanism of ‘identification with the aggressor’ could have served as a source of a feeling of triumph.

### Perversion

Concerning the role of sexuality, the experts assumed that traumatic guilt, which was also associated with post-traumatic conditions, could be maintained at a distance via the defense mechanism of sexualization. Insofar as the ego can sexualize fear, trauma can be controlled (Khan, 1963). The initial physical recovery of the survivors was accompanied by a period of acting out aggressive and sexual phantasies (Klein, 2012b). Category *4) Sexuality as a practical category* shows that gaining power (the feeling of being potent and self-efficient because of the young hero’s liberation from the labor camp) led to sexual arousal. In addition to this general mechanism, which attracts viewers of pornography, individual suffering was distorted in the Stalags and secondarily processed, thereby transforming pain into pleasure and maintaining potency. This is an important difference between consumers of other forms of pornography and strengthens the claim of the influence of a specific social context. The previously suppressed anger that originated, for example, via identification with the aggressor, had the inner psychic function of doing justice to an introjected longing for punishment. It expressed itself in a phantasized (Stalags) or real (Eichmann trial) act of revenge on the torturers and could lead to feelings of liberation through the consumption of the sadomasochistic plot.

As Kernberg (1991, 333-334) puts it, ‘The degree to which the perverse tendencies are transformed into action depends on the predominance of aggressive over libidinal components in the individual’s instinctual equilibrium and the regressive nature of his personality structure, including the regression and/or disintegration of the superego, the predominance of splitting processes in the ego, the consolidation of a pathological grandiose self and the weakening and loss of ego boundaries.’ In simpler terms, the less stable a person’s ability to manage inner conflicts and impulses (due to weaknesses in their ego structure), the more likely it is that they will act on deviant or perverse behaviors. A strong ego helps the person mediate their desires, emotions, and social expectations; however, when the ego is unstable or fragmented, deviant behaviors are more likely to emerge. Another important aspect describes hostility, which is expressed towards intimate relationships in the Stalags insofar as an object (the female guard) is aggressively occupied by rape fantasies. This view corresponds to the modern psychodynamic understanding of paraphilia (perversion), where hostility in the context of intimate relationships is the key component in considering an unusual (deviant) behavior paraphilic (i.e., as an indicator of a deviant pathological sexual preference).

Reiche (2005) describes the criteria that must be fulfilled to define a behavior as perverse. Those were generated for the individual, though they can be applied to the Stalags: the presence of a fetish (also human partial objects like blonde SS guards); the perverted scene (in any case present in the sense of staging the revenge fantasies); addictive unpostponability (craving) in the sense of the Stalags aiming at the periodic repetition of the scene; and a ‘doll-in-the-doll’ setting (in every manifest perversion there is another, repulsed perversion)—the spaces between the dolls consist of unbearable emptiness and black horror; in the sense of the Stalags, these missing links refer to the traumatizations of the Shoah. From a sociopsychological perspective, the act of rape needs to be understood in the framework of sexual scripts, which are influenced by social contexts, such as gendered power imbalances between men and women. Sexual scripts are socially learned guidelines or expectations about how sexual behavior is supposed to unfold in different situations and about who holds the power and has the agency in sexual encounters. Thus, rape is more than simply an abnormal individual act; it is seen as an extreme manifestation of these sexual scripts. As societies have ingrained scripts about gender roles, sexual entitlement, and the dynamics of power and consent in sexual encounters, rape is also a social problem (Gavey, 2018), which is discussed in the following categories of “Intergenerational Conflicts” and “Society.”

### Intergenerational conflicts

Shoah affects the first and subsequent generations through transgenerational trauma transmission (Klein, 2012b). The experts interviewed highlighted the importance and intergenerational function of the Stalags to the end of breaking their parents’ silence in a blatant way by pornographic depictions of conflicts (see Category *2), Sexuality as a Political Category*. Glasser (1979, 1986) defines a so-called core complex, i.e., a conflict between a deep attachment to the mother (loss of identity) and separation from the mother (loneliness, fear). A ‘way out’ of this dilemma is the sexualization of aggression directed against her. The core conflict deals with narcissistic integrity and struggles to find one’s own autonomy. A breaking of the ‘culture of silence’ evolved—beyond the taboo of sexuality in society and family—by rejecting one’s own victim role and taking the perspective of a perpetrator. Further mechanisms in this regard are the defaming of the opponent, the accusing of the parents, and even the addressing of a mother-conflict by the turning of gender roles, as hinted at by a producer of the Stalags in the film by Libsker (2008).

### Society

Categories *5) Reflection of the new ideal of masculinity* and *6) The influence of Hollywood cinema on aesthetics and plot* underline the importance of the political and social context beyond the individual and family context. The readers and producers of the Stalags were strongly influenced by militarized Israeli society, a certain ideal of beauty, and a new description of masculinity. The latter was described by the idea of a young ‘Sabra’: a Jewish young male warrior who fought for his land and Zionist dreams as opposed to (mostly his parents) the ‘weak’ survivors of the Shoah. Although there was a confrontation with the Shoah and feelings of liberation (against their parents) by the second generation via pornographic consumption (Bleimling and Kind, 2015, 24–25), it is important to note that ultimately no real ‘working-through’ (i.e., a successful altercation) of the trauma was achieved. At the same time, while psychoanalytic interpretations offer important insights into the inner dynamics of trauma processing, they represent only one explanatory level of the phenomenon. To avoid reducing the Stalags to individual psychodynamics (depicted in categories 1–3), we see it as essential to contextualize them within broader socio-political and media-historical frameworks. The Stalags must also be understood as products of a specific cultural and generational moment in postwar Israeli society, shaped by Holocaust memory, gendered nationalism, and the visual economies of popular media. To this end, integrating perspectives from cultural trauma theory and memory politics (e.g., Hirsch, 2012; Rothberg, 2009) can help clarify how the Stalags participated in negotiating collective memory, social taboos, and generational conflict through visual and narrative excess (as represented in categories 4–6). Our psychoanalytic reading, therefore, is not intended as a totalizing interpretation but as a critical strand within a broader interdisciplinary analysis of the phenomenon.

## Limitations of the study

This study has provided a broad, interdisciplinary view (including experts from the fields of psychoanalysis, history, forensic science, and sexology) of the Stalag phenomenon. The most relevant critique is that detailed historical work is required. Exact demographic data are still lacking, e.g., regarding the age of the readers of the Stalags at that time, which could provide helpful information about potential transgenerational mechanisms. The limited knowledge of German interviewers of the Stalag phenomenon before the interview must be considered critically. Another methodological concern is that the content of the interviews about the Stalags depended very much on the content presented in the movie of Libsker (which was sent beforehand to the interviewees) and not on the results of scientific research. Further, the experts are not free of bias themselves, which could be shown, e.g., in the category 6 “Influence of the Hollywood cinema on aesthetics and plot,” which can be influenced by the expert’s own research focus or own biographical entanglements, being children of Holocaust survivors.

Furthermore, some ambivalence concerning the extraction of additional categories remained because categories were admitted only by an absolute majority. Other explanations of the Stalags were discussed (e.g., the category “*Liberalization of Sex Morals in Society*”), but they had not been found sufficiently through the interviews, so they were not considered.

### Future research

This study has provided new insights into the sociocultural causes of the Stalag Shoah pornography, focusing on a broad spectrum of explanations ranging from individual psychological defense mechanisms and intergenerational conflicts to societal influences. The question remains unanswered, however, as to why the phenomenon was very limited in time. One reason for the decrease in the popularity and consumption of the Stalags could lie in shame proportional to the enduring Eichmann trial and the induced confrontation with real historical perpetrators and guilty parties. Future research can further investigate the aftermath of Stalagim from the Eichmann trial to contemporary Israel.

## Data Availability

The raw data supporting the conclusions of this article will be made available by the authors, without undue reservation.
